# Supplementary material for: Fine-scale associational effects: Single plant neighbours can alter susceptibility of focal plants to herbivores
Source: PLoS One. 2025 Aug 21;20(8):e0330572. doi: 10.1371/journal.pone.0330572 (PMC12370084; doi:10.1371/journal.pone.0330572)
Supplement: Attachment — Submitted filename: renamed_4fb20.docx [file pone.0330572.s003.docx]

***Response to reviewers – PLOS One PONE-D-25-16194***

**“Fine-scale associational effects: single plant neighbours can alter susceptibility of focal plants to herbivores”**

We thank the two reviewers for providing comment on our manuscript and suggestions for improvement. We have revised the manuscript in response to the reviewers’ queries and suggestions. We have listed every comment raised by each reviewer below, with a statement of how and where the manuscript has been amended in response to these comments, or our justification for making no change. All line numbers referenced below refer to the clean version of the manuscript uploaded.

**Journal Requirements:**

When submitting your revision, we need you to address these additional requirements.

1. Please ensure that your manuscript meets PLOS ONE's style requirements, including those for file naming.

The manuscript meets PLOS ONE’s style requirements.

2. In your Methods section, please provide additional information regarding the permits you obtained for the work. Please ensure you have included the full name of the authority that approved the field site access.

Additional information regarding the permits you obtained for the work have been provided.

**L116 – 119:** *Animal ethics approval was granted by the University of Sydney’s Animal Ethics Committee (protocol number 2022/2196) and scientific license (SL 102186) were obtained from New South Wales Office of Environment and Heritage to undertake the fieldwork.*

3. Thank you for stating the following financial disclosure: [Authors CM and PB acknowledge funding from the Australian Research Council (grant number DP190101441).].

Please state what role the funders took in the study. If the funders had no role, please state: ""The funders had no role in study design, data collection and analysis, decision to publish, or preparation of the manuscript.""

The financial disclosure statement has been removed from the manuscript as per comment 4. below. However in the cover letter and the online Funding Statement section we have amended our wording as such:

*C.M. and P.B.B. acknowledge funding from the Australian Research Council (grant number DP190101441). The funders had no role in study design, data collection and analysis, decision to publish, or preparation of the manuscript.*

Please include this amended Role of Funder statement in your cover letter; we will change the online submission form on your behalf.

As above.

4. Thank you for stating the following in the Acknowledgments Section of your manuscript: [C.M. and P.B.B. acknowledge funding from the Australian Research Council (grant number DP190101441).]

We note that you have provided funding information that is not currently declared in your Funding Statement. However, funding information should not appear in the Acknowledgments section or other areas of your manuscript. We will only publish funding information present in the Funding Statement section of the online submission form.

As above.

Please remove any funding-related text from the manuscript and let us know how you would like to update your Funding Statement. Currently, your Funding Statement reads as follows: [Authors CM and PB acknowledge funding from the Australian Research Council (grant number DP190101441).].

Please include your amended statements within your cover letter; we will change the online submission form on your behalf.

As above.

5. When completing the data availability statement of the submission form, you indicated that you will make your data available on acceptance. We strongly recommend all authors decide on a data sharing plan before acceptance, as the process can be lengthy and hold up publication timelines. Please note that, though access restrictions are acceptable now, your entire data will need to be made freely accessible if your manuscript is accepted for publication. This policy applies to all data except where public deposition would breach compliance with the protocol approved by your research ethics board. If you are unable to adhere to our open data policy, please kindly revise your statement to explain your reasoning and we will seek the editor's input on an exemption. Please be assured that, once you have provided your new statement, the assessment of your exemption will not hold up the peer review process.

The datasets generated during and/or analysed during the current study are currently available in the Sydney eScholarship Repository - <https://hdl.handle.net/2123/33752>.

6. Please include your full ethics statement in the ‘Methods’ section of your manuscript file. In your statement, please include the full name of the IRB or ethics committee who approved or waived your study, as well as whether or not you obtained informed written or verbal consent. If consent was waived for your study, please include this information in your statement as well.

Additional information regarding the permits you obtained for the work have been provided.

**L116 – 119:** *Animal ethics approval was granted by the University of Sydney’s Animal Ethics Committee (protocol number 2022/2196) and scientific license (SL 102186) were obtained from New South Wales Office of Environment and Heritage to undertake the fieldwork.*

7. Please include captions for your Supporting Information files at the end of your manuscript, and update any in-text citations to match accordingly. Please see our Supporting Information guidelines for more information: http://journals.plos.org/plosone/s/supporting-information.

Supporting information has been removed as was R code and data in the oringally submitted manuscript. The datasets generated during and/or analysed during the current study are now currently available in the Sydney eScholarship Repository and hence no longer required as supplementary material - <https://hdl.handle.net/2123/33752>.

8. Please review your reference list to ensure that it is complete and correct. If you have cited papers that have been retracted, please include the rationale for doing so in the manuscript text, or remove these references and replace them with relevant current references. Any changes to the reference list should be mentioned in the rebuttal letter that accompanies your revised manuscript. If you need to cite a retracted article, indicate the article’s retracted status in the References list and also include a citation and full reference for the retraction notice.

Reference list is complete and correct.

**Editor’s comments**

Reviewers offer minor suggestions to improve clarity and coherence, such as refining the introduction by including plant descriptions earlier, adjusting figure legends for clarity, and considering alternative explanations for results based on environmental factors. Additionally, one reviewer recommends replacing numerical notation (e.g., (1), (2)) with clearer wording, while the other suggests defining key terms like "neighborhood" earlier and ensuring Aims are referred to by their specific content rather than labels. Overall, the feedback is highly positive, with both reviewers commending the novelty of the study and its contribution to the field.

We agree with the reviewers’ comments and have gone through and ensured all minor suggested changes have been addressed in the revised manuscript.

**Reviewer #1**

The authors describe a well-designed study that determined that a single unpalatable plant can reduce visitation and browsing of a palatable plant. The methodology and analysis of the experiment are sound and the results are both important and interesting. I just have a few minor comments that I hope will help with the clarity of the manuscript.

We appreciate the reviewer’s comments and suggested corrections. All suggested edits have been included in the revised manuscript.

Introduction: Lines 95-100 The herbivore used for the study, the swamp wallaby, is described but not the plants used. A sentence or two about your three plants selected for the study would be helpful here. That information is provided in material and methods so could just be moved into the introduction.

As suggested, we have now moved the information provided on the three plant species from the materials and methods section into the introduction.

**L101-108:** *To select a low-quality plant species, we conducted haphazard transects in search for a plant that displayed no signs of browsing damage. From these transects we selected Boronia pinnata, a small, highly odorous native shrub species. This species has been previously shown to be avoided by swamp wallabies within the study site [28]. At the study site, B. pinnata plants generally grow alone, and not in clumps with other B. pinnata. We selected two eucalypt species (Eucalyptus punctata and Corymbia gummifera) as higher-quality plants. These were both a known food source of wallabies at the study site (based on [19] and on unpublished work by [29] and [30]). We confirmed the quality of these plants to wallabies as part of the experiment*

Materials and Methods: Lines 124-127: Naturally occurring *B. pinnata* shrubs were used, while nursery grown E*. punctata* and *C. gummifera* were used. You state the size of the nursery grown plants were of similar size but did not give any description of the size of the *B. pinnata* plants chosen. Did you choose *B. pinnata* plants of similar size? If you did please include that information in methods. If similarly sized *B. pinnata* were not chosen that needs to be addressed as a possible limitation to the study.

Thank you for pointing this out. We did record naturally occurring *B. pinnata* size, and yes, they were of similar size to the nursery grown *E. punctata and C. gummifera* we used. We have now included naturally occurring *B. pinnata* size in the revised manuscript (**L129**).

Material and methods, results, and figures: For Aim 1 and 2, you use the term Pair or Treatment and then (1),(2) etc to denote your different Pairs or Treatments. The (#) is often distracting and makes the number seem important rather than just denoting the different pairs or treatments. I suggest removing the () around your numbers. It is especially distracting in the figure legends since there are (a), (b) etc to denote which panel is being described.

Thank you for this suggestion. We agree that the notation was distracting and have now removed all “Pair (1) etc.” and “Treatment (1) etc.” notation throughout the manuscript, including in the Materials and Methods, Results, and figure legends, to aid simplicity and readability.

Figures: Figure 6 legend: lines 294-297 need to describe what panels (a), (b), and (c) denote.

Included

**L306-307:** *Panel a) shows E. punctata and C. gummifera, b) shows E. punctata and B. pinnata, and c) shows C. gummifera and B. pinnata.*

Discussion: Lines 375-387: Reasons for why more of the focal plant *E. punctata* was foraged in Aim 2 versus Aim 1 are discussed here. Is it also possible that the reason more *E. punctate* was foraged on in Aim 2 is that the plots seem to have less undergrowth/browse nearby than the plots in Aim 1. I am basing this off the pictures of the experimental setups in Figures 2 and 3.

Thanks for pointing this out. Aims 1 and 2 were conducted within the same study site, where undergrowth was relatively consistent across all plots. While the photographs in Figures 2 and 3 suggest that there may have been less undergrowth in the plots shown for Aim 2, this is not representative of broader site conditions. We agree this is a valid potential explanation; however, given that undergrowth was similar across the entire site, we do not expect this to have influenced the amount of E. punctata browsing observed between Aims 1 and 2. Unfortunately, we did not take additional photos that better capture the undergrowth in the Aim 2 plots to clarify this point.

**Reviewer #2**

This is an excellent manuscript that sheds new light on the understudied question of how small a plant’s neighborhood can be while still influencing its susceptibility to herbivores. By shifting focus to the smallest spatial scale at which neighborhood effects operate, the authors provide a novel perspective on plant–herbivore interactions. This manuscript was well-written, the experimental design and statistical approaches were robust, and the interpretations of the data appropriate. This manuscript was a delight to review. I have a few general, but minor comments that I think would strengthen the manuscript.

We appreciate the reviewer’s comments and suggested corrections. All suggested edits have been included in the revised manuscript.

A general suggestion: This manuscript has a quite a few moving parts with multiple plant species and combinations and numbers of said species. Given this, it can be hard to keep track of what “Aim 1” vs. “Aim 2” when they are used in a sentence out of context (e.g. L376). While I think it is fine to name them Aim 1 and Aim 2, I suggest referring to them based on their content (i.e., what they are actually testing).

Thank you for this helpful suggestion. We have revised the manuscript to refer to each aim by its content (i.e., the specific experiment being tested) rather than solely as “Aim 1” or “Aim 2” throughout, to improve clarity for readers.

**L140-143:** *Aim 1 (single neighbour experiment) images show a motion-triggered camera above a tub holding (a) an E. punctata seedling (left) next to one C. gummifera seedling (right) and (b) an E. punctata seedling (left) next to one naturally occurring B. pinnata plus pot with soil (right); the C. gummifera and B. pinnata treatment is not shown.*

**L168 – 172***: Aim 2 (neighbourhood experiment) Treatments: (a) a single neighbour of one B. pinnata next to one E. punctata (left), (b) a neighbourhood of five B. pinnata surrounding one E. punctata, (c) an untreated control, one E. punctata on its own and (d) a procedural control, five pots with soil surrounding one E. punctata. Image (c) and (d) show experimental set up of motioned-triggered cameras fastened to wooden stakes. Focal plants are circles in yellow.*

**L388-397:** *For the single neighbour experiment (Aim 1, which tested whether a single plant neighbour elicits an associational effect), high-quality focal plants were browsed less by wallabies when next to a single low-quality neighbour. In contrast, in the neighbourhood experiment (Aim 2, which tested whether multiple neighbours elicit stronger associational effects), if browsed, high-quality focal E. punctata were almost always completely consumed regardless of Treatment. This difference in browsing severity may reflect a difference in focal E. punctata quality [8, 40] and/or a difference in B.pinnata quality between the two experiemnts. Although from the same nursery, E. punctata seedlings were purchased at different times within a year and possibly grown under different conditions. Similarly, B. pinnata was naturally occurring in Aim 1 but nursery-grown in Aim 2. Different abiotic growing conditions could affect nutrient and plant secondary metabolite concentrations (e.g. [43]).*

The opening paragraph is well-written, however, I think a few tweaks to it might strengthen the foundational ideas for the rest of the manuscript. For example:

L43: I think it might be helpful to define a neighborhood (in your context) here, given that it is a term heavily used through the manuscript. For example, it might be useful to say something along the lines of “The assemblage of plants in a patch forms a neighborhood, which can…”

Noted and updated.
**L43-45:** *The assemblage of plants in a patch forms a neighbourhood. This plant neighbourhood can influence the susceptibility of an individual plant to being found and eaten by herbivores, known as an associational effect [1-5].*

L46: I suggest adding an additional sentence between the ones that start and end on L46 to set up the idea that the neighborhood can either have a positive or negative effect on a given plant. The sentence beginning with 'When herbivores…' could be revised to start with 'For example, when herbivores…,' which would create a smoother transition and more clearly set up the contrast with the following sentence that begins 'In contrast…,' highlighting the opposing effects of a neighborhood.

Thank you for this suggestion, noted and updated.

**L45-51:** *Associational effects are often a consequence of herbivores seeking high-quality ‘palatable’ patches and avoiding low-quality patches [6-9] to maximise their foraging efficiency. For example, when herbivores select among patches, a focal plant in a high-quality patch is more likely to be visited by a herbivore hence more susceptible to being consumed, known as associational plant susceptibility [10-13]. In contrast, a focal plant in a low-quality patch (e.g., a high density of low nutrient, structurally, and/or chemically defended species) that is avoided by herbivores will be safe from being eaten, known as associational plant refuge [1, 2, 14, 15].*

In the Statistical Analysis section:

L185: Is it possible to include this information elsewhere to omit an orphan paragraph?

We have moved this sentence to the end of the Statistical Analysis section to omit to orphan paragraph (**L223-224 and L242-244)**.

In the Discussion:

L368-374: Might it be possible that more plants could emit a strong smell that might be detectable from larger distances? Ultimately, this could influence the distance at which decisions could be made rather than the choice itself.

Thank you for this comment. We have revised the manuscript to incorporate this point, noting that a greater number of *B. pinnata* plants could produce stronger odour cues detectable from greater distances, potentially influencing the distance at which wallabies make foraging decisions rather than their ultimate choice to forage.

**L379-383:** *Nevertheless, although a single B. pinnata plant may have the same effect on wallabies’ ultimate foraging choice as five plants, having five B. pinnata plants would likely emit a stronger quantitative odour cue that could be detected from greater distances, potentially influencing how far away wallabies make their foraging decisions, rather than changing their choice itself, or possibly masking the odour of focal plants altogether.*
